# Diversification of the AlpB Outer Membrane Protein of Helicobacter pylori Affects Biofilm Formation and Cellular Adhesion

**DOI:** 10.1128/JB.00729-16

**Published:** 2017-02-28

**Authors:** Hideo Yonezawa, Takako Osaki, Toshiyuki Fukutomi, Tomoko Hanawa, Satoshi Kurata, Cynthia Zaman, Fuhito Hojo, Shigeru Kamiya

**Affiliations:** a Department of Infectious Diseases, Kyorin University School of Medicine, Tokyo, Japan; b Department of Pharmacology, Kyorin University School of Medicine, Tokyo, Japan; c Institute of Laboratory Animals, Graduate School of Medicine, Kyorin University, Tokyo, Japan; Geisel School of Medicine at Dartmouth

**Keywords:** Helicobacter pylori, biofilms

## Abstract

Helicobacter pylori is one of the most common causes of bacterial infection in humans, and it forms biofilms on human gastric mucosal epithelium as well as on *in vitro* abiotic surfaces. Bacterial biofilm is critical not only for environmental survival but also for successful infection. We previously demonstrated that strain TK1402, which was isolated from a Japanese patient with duodenal and gastric ulcers, has high biofilm-forming ability *in vitro* relative to other strains. In addition, we showed that outer membrane vesicles (OMV) play an important role in biofilm formation. The aim of this study was to analyze which protein(s) in the OMV contributes to biofilm formation in TK1402. We obtained a spontaneous mutant strain derived from TK1402 lacking biofilm-forming ability. The protein profiles of the OMV were compared between this mutant strain and the wild type, and it was found that AlpB, an outer membrane protein in the OMV of the mutant strain, was markedly decreased compared to that of the wild type. Restoration of TK1402 *alpB* to the mutant strain fully recovered the ability to form biofilm. However, restoration with *alpB* from other strains demonstrated incomplete recovery of biofilm-forming ability. We therefore inferred that the variable region of AlpB (amino acid positions 121 to 146) was involved in TK1402 biofilm formation. In addition, diversification of the AlpB sequence was shown to affect the ability to adhere to AGS cells. These results demonstrate a new insight into the molecular mechanisms of host colonization by H. pylori.

**IMPORTANCE** Bacterial biofilm is critical not only for environmental survival but also for successful infection. The mechanism of Helicobacter pylori adherence to host cells mediated by cell surface adhesins has been the focus of many studies, but little is known regarding factors involved in H. pylori biofilm formation. Our study demonstrated that AlpB plays an important role in biofilm formation and that this property depends upon the specific sequence of *alpB*. This in turn was shown to be important in the ability to adhere to gastric cells. We anticipate that these results will provide new insight into the molecular mechanisms of H. pylori colonization.

## INTRODUCTION

Helicobacter pylori is a spiral, microaerophilic, Gram-negative bacterium that colonizes the human gastrointestinal tract, primarily the stomach (1). H. pylori persists lifelong within the human stomach and is estimated to infect the stomachs of approximately half of the world's population. Although almost all individuals infected with H. pylori persist as nonsymptomatic carriers for their lifetimes, in some, H. pylori can cause gastritis and peptic ulcers (2, 3). More severe diseases, such as mucosa-associated lymphoid tissue (MALT) lymphoma and gastric adenocarcinoma, are also associated with infection (4). Analysis using gastric biopsy shows that H. pylori is partially found deep in the mucous layer, with remaining bacteria attached to the surface of gastric epithelial cells (5).

Bacterial biofilms are surface-attached microorganism communities. Biofilm formation is critical not only for environmental survival but also for successful infection in numerous pathogenic bacteria (6–8). Biofilm formation is often observed in bacteria that colonize or infect humans or animals. Some studies have alluded to the ability of H. pylori to form biofilms *in vitro* (9–11). In addition, H. pylori can exist in biofilms formed on the surface of human gastric mucosa (12–14). Recently, several researchers have reported on the characteristics of the H. pylori biofilm (15–17). We previously demonstrated that strain TK1402, isolated from a Japanese patient with duodenal and gastric ulcers, had high biofilm-forming ability *in vitro* (18, 19), demonstrated by the relative thickness of the biofilm. The outer membrane vesicles (OMV) released by TK1402 play an important role in biofilm formation as extracellular matrix components (18, 20). However, the factors involved in biofilm formation within the bacterial cell remain poorly understood.

Adherence is important for bacterial infection of a host. Bacterial adherence to host cells is mediated by adhesins on the bacterial cell surface. The components of H. pylori adhesins have been the focus of many studies (21). One of them, two adjacent homologue genes, *alpA-alpB*, annotated as *omp20-omp21* and *hopC-hopB* in the 26695 and G_2_7 genomes, respectively, were first implicated in adhesion by Odenbreit et al. (22). They reported that *alpA-alpB* knockout mutant strains were defective in adherence to human gastric tissue sections. In addition, subsequent studies demonstrated that the AlpA and AlpB proteins were found to exist ubiquitously in H. pylori strains and were required for gastric colonization in the guinea pig stomach (23, 24). Lu et al. reported that AlpA and AlpB could induce gastric injury by mediating adherence to gastric epithelial cells (25). In addition, Senkovich et al. demonstrated that both AlpA and AlpB contribute to laminin binding (26).

A proportion of H. pylori-infected people develop symptomatic diseases, but all of the infected people, including asymptomatic carriers, are a high-risk population for gastric adenocarcinoma and MALT lymphoma. Accordingly, it is recommended that H. pylori-infected patients receive eradication therapy even if they are asymptomatic. Eradication therapy demonstrates a high success rate despite the variety of antibiotics used. However, failure of eradication therapy is an increasing problem. The major cause of eradication failure is thought to be the existence of antibiotic-resistant H. pylori (27–29). On the other hand, several reports indicate that the biofilm formation of H. pylori influences eradication therapy (30, 31), and we have previously demonstrated that biofilm formation of H. pylori TK1402 increases resistance to clarithromycin (CAM) and can affect CAM resistance mutation generation in an *in vitro* model (32). Recently, Gaddy et al. demonstrated that biofilm formation by H. pylori could resist the antimicrobial activity of calprotectin via lipid A modification (33). On the basis of these observations, studies regarding the biofilm formation of H. pylori are of increasing importance.

Here, we identify a factor which plays an important role in the biofilm formation of TK1402, comparing wild-type OMV protein profiles to those that are produced by a spontaneous weak biofilm-forming mutant strain derived from TK1402. We demonstrate that AlpB plays a critical role in biofilm formation in TK1402. The amino acid sequence of AlpB indicates that almost all amino acid sequences are conserved; however, a variable region was shown to be associated with biofilm formation by *alpB* restoration. In addition, the genetic diversity of AlpB was shown to contribute to adherence of H. pylori to AGS cells.

## RESULTS

### Identification of a factor involved in TK1402 biofilm formation.

**FIG 1:**
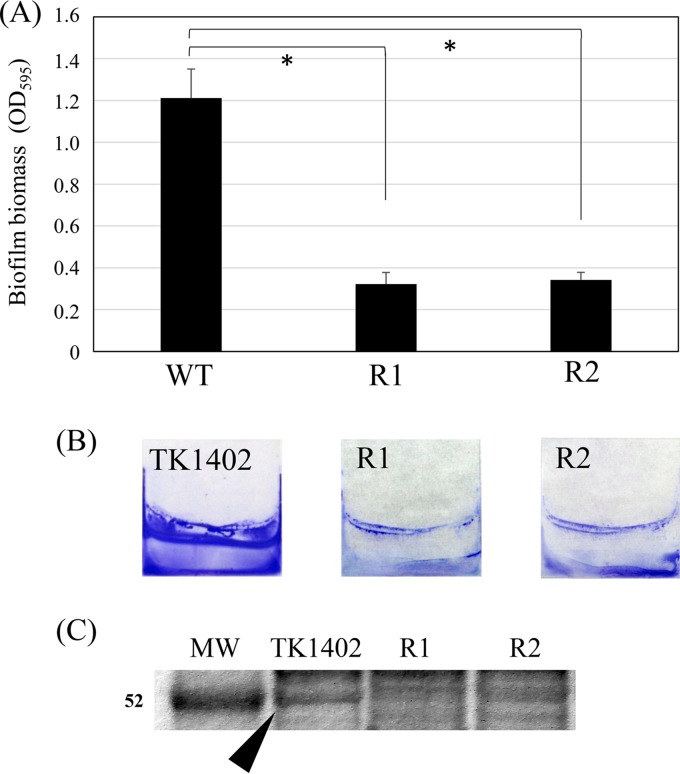
Biofilm formation by wild-type TK1402 (WT) and two spontaneous TK1402 CAM-resistant strains, TK1402CAMR1 (R1) and TK1402CAMR2 (R2). (A) H. pylori biofilms formed on glass coverslips after 3 days were quantified by crystal violet (CV) staining and ethanol elution. The result is expressed as the means ± 1 standard deviation from at least three independent experiments. (B) CV-stained biofilm of H. pylori strains grown on the surfaces of glass coverslips in brucella-FCS broth. (C) Protein profiles of OMV from wild-type TK1402 (TK1402), TK1402CAMR1 (R1), and TK1402CAMR2 (R2). The approximate position of the 52-kDa band in the wild type is shown by an arrowhead. An asterisk indicates significant difference (*P* < 0.05) relative to the level of wild-type biofilm biomass. MW, molecular weight in thousands.

In our previous study, we obtained 54 spontaneous CAM-resistant mutant strains derived from TK1402 (32). These mutant strains have at least a point mutation at either position 2142 (43 strains) or 2143 (11 strains) of the 23S rRNA gene (32). We randomly picked 8 strains (4 strains have a 2142 mutation and 4 strains have a 2143 mutation; all of the strains have adenine-to-guanine transversion), and the biofilm-forming abilities of these mutant strains were analyzed. Six strains exhibited biofilm-forming abilities similar to that of the wild type (data not shown). However, two mutant strains, named TK1402CAMR1 and TK1402CAMR2, showed significantly lower levels of biofilm formation than the wild type (Fig. 1A). The crystal violet (CV)-stained biofilms of the wild type and the mutant strains are shown in Fig. 1B. Since we previously demonstrated that OMV released by TK1402 play an important role in the biofilm extracellular matrix (18), we isolated the OMV from wild-type and mutant strains and analyzed their protein profiles. The 52-kDa protein band seemed to be weaker in the OMV from the mutant strains (Fig. 1C) than from the wild-type strain. In order to clarify differences of band intensity between wild-type and mutant strains, the 52-kDa band densities were evaluated using ImageQuant LAS-4000 (GE Healthcare). The band intensity of the mutant strains was clearly decreased compared to that of the wild type, with relative values of approximately 0.23 in TK1402CAMR1 and 0.43 in TK1402CAMR2; the value for the wild type was set to 1.0. The 52-kDa protein was then analyzed with mass spectrometry and identified as AlpB (coverage rate, 46.29%; number of unique peptides, 1; score, 1,298.77).

### AlpB plays an important role in biofilm formation by TK1402.

**FIG 2:**
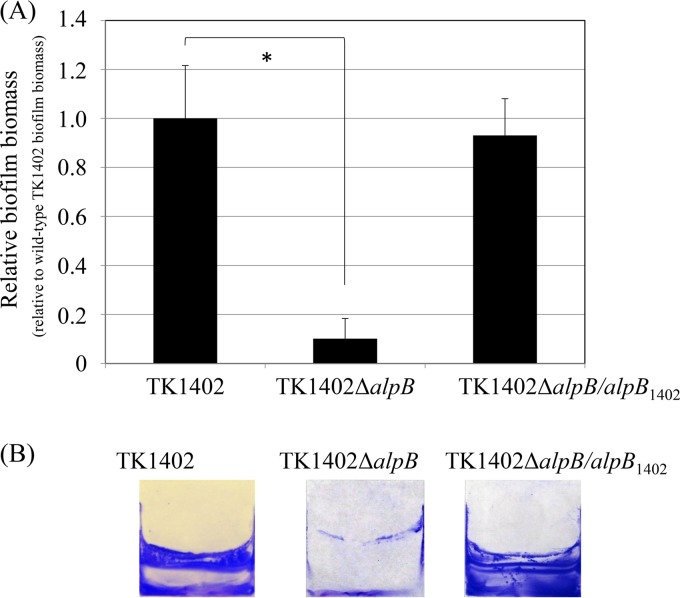
Biofilm formation by wild-type TK1402 (TK1402), its isogenic *alpB* mutant strain (TK1402Δ*alpB*), and a TK1402 *alpB* restored strain (TK1402Δ*alpB/alpB*_1402_). (A) Quantification of H. pylori biofilms formed on glass coverslips after 3 days by CV staining and ethanol elution. The data are expressed as the means ± 1 standard deviation from at least three independent experiments. (B) CV-stained biofilm of wild-type TK1402 (TK1402), TK1402Δ*alpB*, and TK1402Δ*alpB/alpB*_1402_ strains of H. pylori grown on the surfaces of glass coverslips in brucella-FCS broth. An asterisk indicates significant difference (*P* < 0.05) relative to the level of wild-type biofilm biomass.

In order to analyze whether AlpB was involved in biofilm formation by TK1402, an *alpB*-deficient mutant (TK1402Δ*alpB*) strain was constructed and biofilm assay was carried out. The wild-type and TK1402Δ*alpB* strains exhibited similar growth in brucella broth supplemented with 7% fetal calf serum (brucella-FCS broth) (data not shown). In biofilm formation, the TK1402Δ*alpB* strain showed significantly lower activity than the wild type (Fig. 2A). The CV-stained biofilms of wild-type and TK1402Δ*alpB* strains on the coverslip surface are shown in Fig. 2B. In the *alpB* restoration (TK1402Δ*alpB/alpB*_1402_) strain, the biofilm-forming ability was recovered to the wild-type level (Fig. 2). These results confirm that AlpB plays an important role in biofilm formation by TK1402.

### Expression of *alpB* gene.

**FIG 3:**
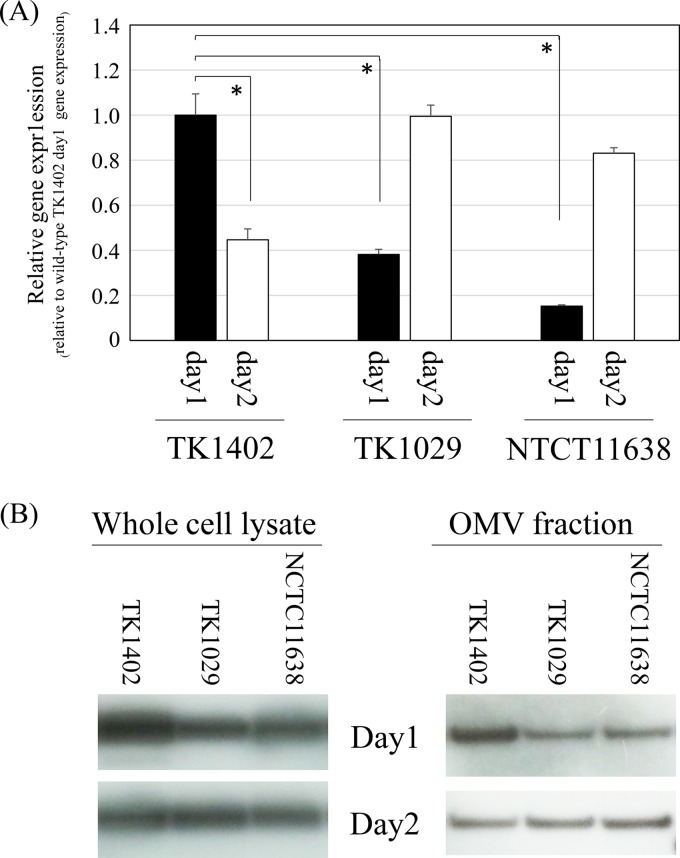
Analysis of *alpB* gene expression in H. pylori strains TK1402, TK1029, and NCTC11638. (A) The quantity of cDNA corresponding to the *alpB* gene was determined by real-time PCR and was normalized to that for the 16S rRNA gene. The experiment was repeated three times with at least duplicate samples from independently isolated RNA preparations, and data are expressed as the means from all of the experiments ± 1 standard deviation. An asterisk indicates significant difference (*P* < 0.05) relative to the mRNA expression level (for TK1402 on day 1 versus TK1402 on day 2, TK1029 on day 1, or NCTC11638 on day 1). (B) A FLAG epitope was inserted into chromosomal *alpB* at the 3′ terminus in each H. pylori strain, and Western blotting with anti-FLAG monoclonal antibody M2 was performed with cell lysates or OMV of each H. pylori strain.

Odenbreit et al. demonstrated that the AlpB proteins are expressed in 200 clinical isolates (23). In addition, we confirmed that the *alpB* sequences are present in all of the H. pylori genomes already deposited in GenBank. Based on these results, we hypothesized that *alpB* of TK1402 has a specific characteristic(s) different from that of other strains. First, we determined whether there were differences in the level of transcription of the *alpB* gene between TK1402 and other strains by quantitative real-time reverse transcription-PCR (RT-PCR). RNA samples from strain TK1402, TK1029, or NCTC11638, cultured for 24 h and 48 h, were extracted, and *alpB* gene expression was analyzed (Fig. 3A). A comparison of *alpB* gene expression in these strains after 24 h of culture revealed that the expression of the gene was significantly elevated in TK1402 compared to the level in the other strains. However, *alpB* expression in TK1402 decreased after 48 h of culture. On the other hand, expression levels of *alpB* in TK1029 and NCTC11638 cultured for 48 h increased to a similar level as the expression of *alpB* in TK1402 cultured for 24 h. We next examined the level of gene product in these strains, but anti-AlpB antibody was not available. Thus, we inserted FLAG epitope sequences into chromosomal *alpB* at the 3′ terminus as described in Materials and Methods, and the amounts of AlpB-FLAG in whole-cell lysate or OMV were analyzed using Western blotting with anti-FLAG monoclonal antibody M2. The amount of AlpB-FLAG production was found to be increased in both cell lysate and OMV of 24-h-cultured TK1402 compared to other strains (Fig. 3B). However, the amounts of AlpB-FLAG were equal in all of the 48-h-cultured strains. This result was consistent with the result of the *alpB* gene expression analysis. These results suggest that the rapid expression of AlpB during early culture contributes to the high biofilm-forming ability of TK1402. However, it is unlikely that the difference in the biofilm-forming ability between TK1402 and other strains can be completely explained by this result.

### Sequence analysis of *alpB* and restoration with *alpB* genes from other strains.

**FIG 4:**
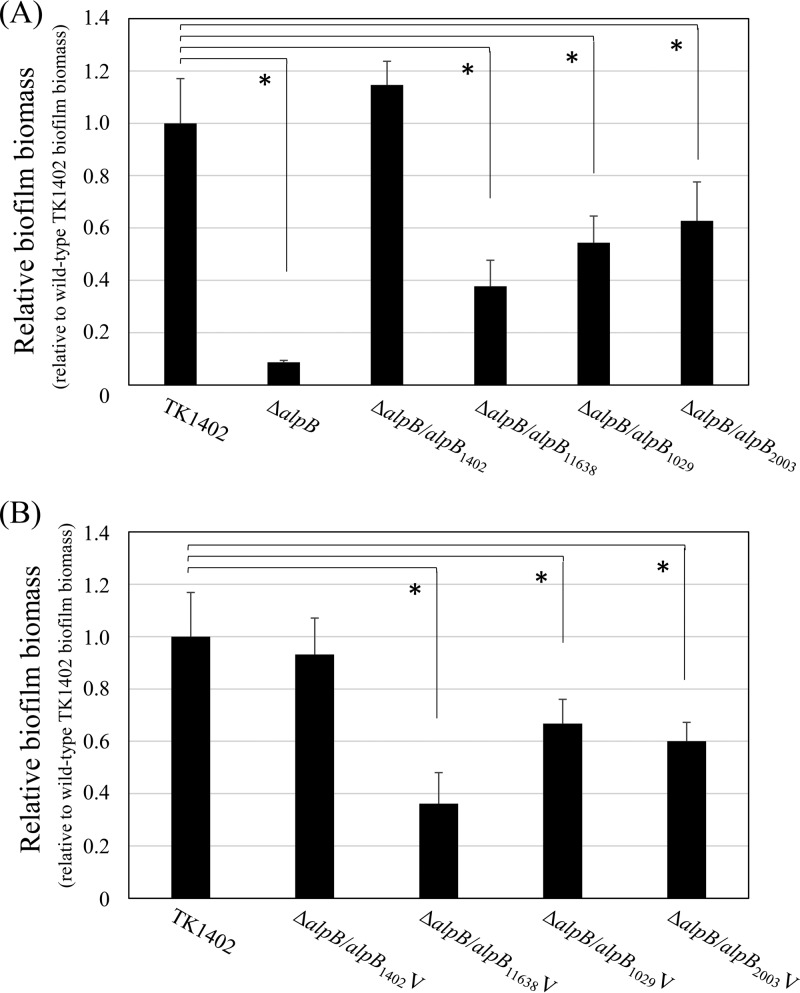
Biofilm formation by wild-type TK1402 (TK1402) and the *alpB* strains restored with *alpB* from other strains. (A) Intact *alpB* from strains TK1402, NCTC11638, TK1029, and KR2003 was introduced into a TK1402Δ*alpB* strain (TK1402Δ*alpB/alpB*_1402_, TK1402Δ*alpB/alpB*_11638_, TK1402Δ*alpB/alpB*_1029_, or TK1402Δ*alpB/alpB*_2003_), and biofilm-forming ability was analyzed. (B) The *alpB* variable region from strains TK1402, NCTC11638, TK1029, and KR2003 was inserted into the TK1402 *alpB* region and introduced into a TK1402Δ*alpB* strain (TK1402Δ*alpB/alpB*_1402_V, TK1402Δ*alpB/alpB*_11638_V, TK1402Δ*alpB/alpB*_1029_V, or TK1402Δ*alpB/alpB*_2003_V). The biofilm-forming abilities of these restored strains were analyzed. Biofilm quantity was calculated relative to that for wild-type TK1402, which was set to 1.0. All of the results were expressed as the means ± 1 standard deviation from at least three independent experiments. An asterisk indicates significant difference (*P* < 0.05) relative to the level of wild-type biofilm biomass.

Lu et al. demonstrated that almost all of the *alpB* sequence is conserved, save for a small variable region. In particular, no strain shows an identical sequence between amino acid positions 121 and 146. We analyzed the AlpB amino acid sequences of the strains used in this study (see Fig. S1 in the supplemental material). As expected, the variable region of all strains showed different sequences (Fig. S1, underlining). Therefore, we inferred that the specific sequences of this region in TK1402 are associated with its biofilm formation ability. Thus, we constructed restored strains of the TK1402Δ*alpB* strain with *alpB* of other strains (NCTC11638, TK1029 or KR2003; TK1402Δ*alpB/alpB*_11638_, TK1402Δ*alpB/alpB*_1029_, and TK1402Δ*alpB/alpB*_2003_ strains, respectively), and biofilm-forming ability was assessed. Contrary to expectations, the biofilm-forming ability of these restored strains did recover compared to the original TK1402Δ*alpB* mutant strain (Fig. 4A). However, the recovery was incomplete and significantly decreased compared with the wild-type or TK1402Δ*alpB/alpB*_1402_ strain (Fig. 4A); for example, the TK1402Δ*alpB/alpB*_11638_ strain restored about 40% biofilm-forming ability compared to the wild type. We next constructed strains which restored the TK1402Δ*alpB* strain with a fragment containing the variable region of *alpB* only (gene positions 330 to 522, corresponding to amino acid positions 110 to 174 [Fig. S1, box]). The same strains were used (TK1402, NCTC11638, TK1029, and KR2003; TK1402Δ*alpB/alpB*_1402_V, TK1402Δ*alpB/alpB*_11638_V, TK1402Δ*alpB/alpB*_1029_V, and TK1402Δ*alpB/alpB*_2003_V strains, respectively), and biofilm assays were carried out. The results were similar to those for the intact *alpB* restored strain (Fig. 4B). These results indicated that *alpB*, especially the variable region of the protein, plays an important role in TK1402 biofilm formation.

**FIG 5:**
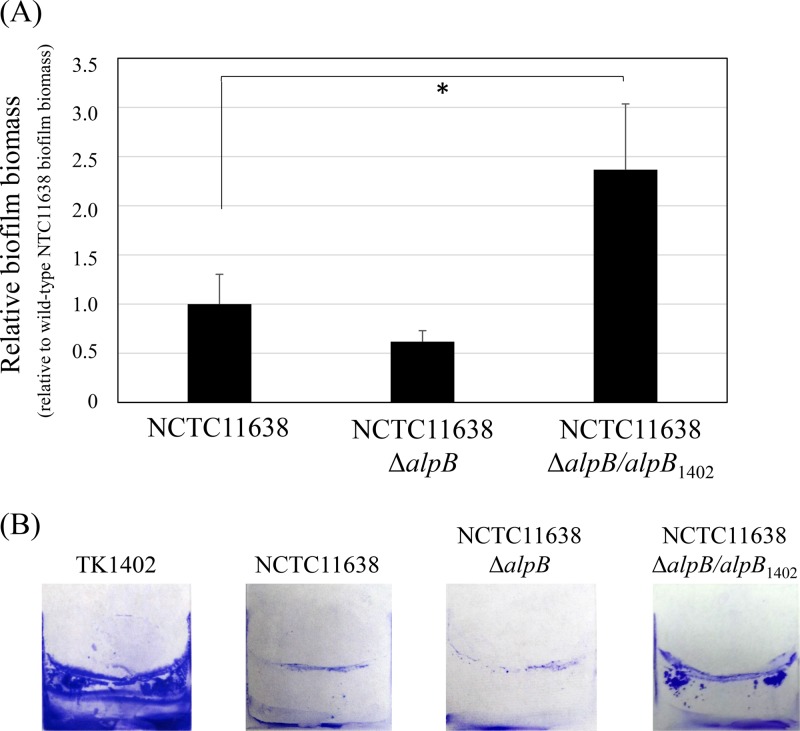
Biofilm formation by wild-type NCTC11638, an *alpB* mutant strain (NCTC11638Δ*alpB*), and NCTC11638Δ*alpB* with TK1402 *alpB* restored (NCTC11638Δ*alpB/alpB*_1402_). (A) Intact *alpB* from strain TK1402 was restored into the NCTC11638Δ*alpB* strain, and biofilm-forming ability was analyzed. The results were expressed as the means ± 1 standard deviation from at least three independent experiments. (B) CV-stained biofilms of wild-type TK1402 (TK1402), wild-type NCTC11638 (NCTC11638), and the NCTC11638Δ*alpB* and NCTC11638Δ*alpB/alpB*_1402_ strains grown on the surfaces of glass coverslips in brucella-FCS. An asterisk indicates significant difference (*P* < 0.05) relative to the level of wild-type NCTC11638 biofilm biomass.

We next constructed an NCTC11638 *alpB*-deficient mutant (NCTC11638Δ*alpB*) strain. Strain NCTC11638 formed relatively little biofilm compared to TK1402 (18) and relatively weaker biofilm formation than wild-type NCTC11638 (Fig. 5A). The NCTC11638Δ*alpB/alpB*_1402_ strain, which restored the NCTC11638Δ*alpB* strain with TK1402 *alpB*, formed significantly thicker biofilms than the NCTC11638 wild-type or NCTC11638Δ*alpB* strain (Fig. 5A). The CV-stained biofilms of these strains are shown in Fig. 5B. However, biofilm formation of the NCTC11638Δ*alpB/alpB*_1402_ strain was clearly weaker than that of the TK1402 wild type (Fig. 5B). These results indicate that although *alpB* is one of the factors responsible for the exceptional biofilm formation ability of TK1402, other factors are likely also involved.

### Differences in the adhesion to AGS cells between restoration strains.

**FIG 6:**
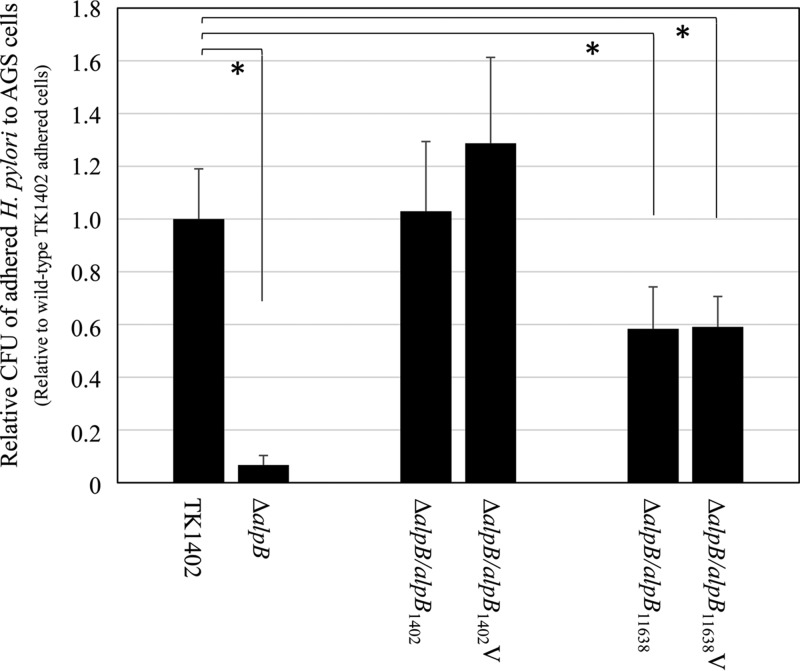
Adherence of H. pylori TK1402 wild type, the TK1402Δ*alpB* mutant strain (Δ*alpB*), and the restored strains to AGS cells. Intact *alpB* or the variable region of *alpB* from strain TK1402 or NCTC11638 was restored in the TK1402Δ*alpB* strain, and the adherence of the resulting strains (TK1402Δ*alpB/alpB*_1402_, TK1402Δ*alpB/alpB*_1402_V, TK1402Δ*alpB/alpB*_11638_, and TK1402Δ*alpB/alpB*_11638_V) to AGS cells was analyzed with CFU counts. All of the results were expressed as the means ± 1 standard deviation from at least three independent experiments. An asterisk indicates significant difference (*P* < 0.05) relative to the level of the wild type.

Odenbreit et al. indicated that AlpB in H. pylori is involved in the ability to adhere to human gastric tissue (22). In addition, Senkovich et al. also demonstrated that the *alpB* locus contributes to adherence of H. pylori to AGS gastric cell lines (26). In order to examine the participation of the various AlpB proteins in the adherence to human gastric cells, we evaluated the adhesion abilities of wild-type TK1402 and its mutant strains by determining the number of bacterial cells adhered to AGS gastric cells using CFU counts. Since TK1402Δ*alpB/alpB*_11638_ and TK1402Δ*alpB/alpB*_11638_V strains, which were constructed using NCTC11638 *alpB*, exhibited the lowest biofilm-forming ability, these strains were analyzed for cell binding ability. The TK1402Δ*alpB* strain exhibited lower-level binding to AGS cells than wild-type TK1402, and this result was consistent with previous reports (Fig. 6) (22, 26). In TK1402Δ*alpB/alpB*_1402_ and TK1402Δ*alpB/alpB*_1402_V strains, which were restored with TK1402 *alpB* (Fig. 6), the adhered bacterial numbers were similar to those of the wild type. On the other hand, TK1402Δ*alpB/alpB*_11638_ and TK1402Δ*alpB/alpB*_11638_V strains exhibited significantly decreased attachment to AGS cells compared to the wild type (Fig. 6). These results indicated that the variation in AlpB amino acid sequence affects adherence of H. pylori to AGS cells.

## DISCUSSION

Gram-negative bacteria continuously shed OMV from the cell surface. OMV contain periplasmic and cytoplasmic proteins, DNA and RNA, and outer membrane proteins (34, 35). The OMV is considered to be part of the secretion system that plays an important role in delivering toxin (35). Furthermore, OMV have important functions in cell-cell interactions as well as biofilm formation (36). In H. pylori, Olofsson et al. reported that the biological role of OMV is in toxin delivery (37). In addition, we previously demonstrated that OMV play an important role in the extracellular matrix of biofilm formation in H. pylori strain TK1402 (18). Further investigation of the factors resulting in the high biofilm-forming ability of this strain is critical for understanding the mechanism of H. pylori biofilm formation. In the present study, we identified AlpB as an important protein for biofilm formation from the results of a comparison of OMV protein profiles (Fig. 1C) between TK1402 and its spontaneous weak biofilm-forming mutant strains (Fig. 1A and B).

The *alpB* mutant strain displayed significantly decreased biofilm formation compared to wild-type TK1402 (Fig. 2). Odenbreit et al. demonstrated that all isolates of H. pylori produced AlpB protein (23). These results suggested that TK1402 AlpB has a unique property associated with biofilm formation. We first analyzed the differences in *alpB* expression between the strains. Although a difference of expression pattern was found between TK1402 and other strains, the expression level did not seem to be the only factor involved (Fig. 3). The development of TK1402 biofilm took 3 days for maturation under these conditions (18). High expression of AlpB in the initial stage may contribute to the high biofilm-forming ability of TK1402, but we suspected that a different property of TK1402 AlpB also was involved in biofilm formation.

H. pylori is known to have a high degree of genome diversity due to a high frequency of mutation and recombination (38–41). Recently, we reported on multilocus sequence typing and whole-genome sequence analyses of very closely related H. pylori strains from the same family members, consisting of parents and children in Japan, suggesting adaptation to a new host through mutations in virulence-related genes and restriction-modification genes as well as OMP genes, including *alpB* (42, 43). Furthermore, Lu et al. showed that a variable region is present in the *alpB* sequence (25). In particular, there is extreme variation between amino acid positions 121 to 146 of AlpB between H. pylori strains (see Fig. S1 in the supplemental material). In this study, we constructed *alpB* restoration strains containing intact *alpB* or an *alpB* variable region from other strains. The biofilm-forming abilities of these strains were recovered compared to that of the TK1402 *alpB* mutant strain. However, the recovery was only partial compared to wild-type or TK1402 *alpB* restored strains (Fig. 4). Furthermore, the TK1402 *alpB* restored NCTC11638 *alpB* mutant strain showed increased biofilm-forming ability relative to wild-type NCTC11638 (Fig. 5). These results indicated that the variable region of *alpB*, as well as the intact *alpB* of TK1402, plays an important role in biofilm formation. On the other hand, the observation that the biofilm-forming ability of the NCTC11638Δ*alpB/alpB*_1402_ strain (NCTC11638 *alpB* mutant strain with TK1402 *alpB*) was clearly weaker than that of wild-type TK1402 indicates that although *alpB* is important, it is not the only factor affecting TK1402 biofilm formation. Further studies are in progress to investigate these other factors.

Development of bacterial biofilms proceed by initial attachment, microcolony formation, and maturation by mechanisms such as cell-cell attachment (44–46). Senkovich et al. showed that the *alpB* mutant strain derived from strain 26695m could not cause cell aggregation (26). In the present study, TK1402 *alpB* mutant strains also could not exhibit the cell aggregation phenotype (data not shown), suggesting that the *alpB* mutant strain had lost the ability of cell-cell attachment, which may partially explain the decrease in biofilm-forming ability. In addition, restored strains with NCTC11638 and intact *alpB* or a variable region of *alpB* showed significantly lower binding to AGS than the wild type (Fig. 6). These findings suggested that *alpB*, especially the variable region, regulates the attachment to various substrates. It is possible that further investigation of the variable region of AlpB will provide insights into biofilm formation and cell adhesion and clarify how the bacteria colonize human gastric mucosa.

**FIG 7:**
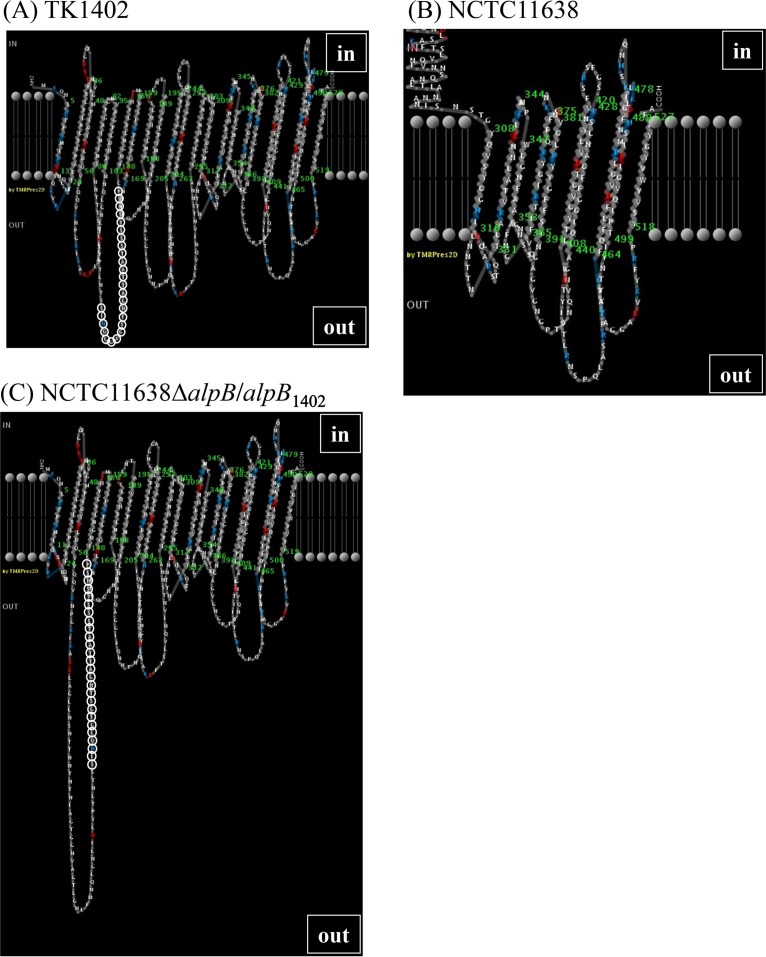
Two-dimensional structure of AlpB from TK1402 (A), NCTC11638 (B), and NCTC11638Δ*alpB/alpB*_1402_ (C) strains based on the prediction by PRED-TMBB. The amino acids of the variable region are shown in open circles, except for NCTC11638. In this strain, the variable region was located further upstream than the displayed amino acids, in an intracellular location, and therefore is not shown in this figure. The NCTC11638Δ*alpB/alpB*_1402_ strain was a restored strain derived from the NCTC11638 *alpB* mutant restored with TK1402 *alpB*.

Since the variable sequence of AlpB is involved in biofilm formation and cell adhesion, it was predicted that the amino acids of the variable region were more likely to localize to the cell surface. In order to analyze the position of the variable region in the structure of AlpB, the two-dimensional (2D) structure of AlpB was analyzed using PRED-TMBB (http://biophysics.biol.uoa.gr/PRED-TMBB/), and models of predicted 2D structure were produced (47). As expected, the variable region was located in an extracellular loop in TK1402 (Fig. 7A; the variable-region amino acids are shown with open circles), but the localization of the variable region in NCTC11638 was inside the outer membrane (Fig. 7B; no variable region is shown, since the amino acids of the variable region are located much further upstream than the displayed residues). The positions of the TK1029 and KR2003 variable regions were similar to that of TK1402 (Fig. S2A and B), and when the variable region of TK1402 was replaced with that of TK1029 or KR2003, its position was identical to that of wild-type TK1402 (Fig. S2C and D). Interestingly, the predicted topology model for the NCTC11638Δ*alpB/alpB*_1402_ restored strain (NCTC11638 *alpB* mutant strain with TK1402 *alpB*) showed a different pattern compared to TK1402 or wild-type NCTC11638 strains (Fig. 7C). The amino acids of the variable region derived from TK1402 appeared in an external loop, suggesting that this region is involved in biofilm formation. However, we did not obtain direct evidence that the variable region of TK1402 AlpB is extracellular. Further analysis will be required to resolve this issue.

Strains TK1402CAMR1 and TK1402CAMR2 are CAM-resistant strains and have at least the point mutations A2142G and A2143G, respectively, in the 23S rRNA (32). Interestingly, although these mutant strains were generated by independently performed experiments (TK1402CAMR1 was derived from biofilm cells, and TK1402CAMR2 was derived from planktonic cells), the protein profiles of the OMV of the mutant strains were similar (data not shown), but their band patterns were different from those of the wild type (Fig. 1D). We recently demonstrated that nucleotide substitution in *alpB* occurs frequently in members of the same family in Japan (42). However, we did not detect nucleotide substitution in the promoter–*alpA-alpB* region of the mutant strains compared to the wild type. The results suggested that mutation(s) occurs in the regulatory system, affecting the expression of proteins, including AlpB, in these mutant strains. We analyzed expression of *alpB* mRNA using TK1402CAMR1 and TK1402CAMR2 for 24 h and 48 h, and the transcriptional level in the mutant strains was similar to that in the wild type (data not shown). This suggested that another factor outside the transcription level suppressed the expression of AlpB in the mutant strains, such as posttranscriptional regulation. On the other hand, Servetas et al. reported that ArsRS, a two-component acid response system in H. pylori, controls biofilm formation (49). Previous reports showed that the ArsRS system regulates the expression of numerous OMPs (48, 50), and therefore the observed decrease in biofilm formation exhibited in TK1402CAMR1 and TK1402CAMR2 may be due to mutation of ArsRS, resulting in repression of the expression of AlpB. Further elucidation of the mutation site in TK1402CAMR1 and TK1402CAMR2 and the regulatory pathway of AlpB expression could help further unravel the mechanism of biofilm formation in TK1402.

In summary, the present study of *alpB* disruption and restoration by *alpB* from other strains has demonstrated that AlpB plays a considerable part in biofilm development in TK1402. Sequence variation of *alpB* affects biofilm-forming ability as well as adhesion to gastric cells. Further analysis of the differences in function afforded by the variable sequence of *alpB* may provide further insight into the colonization mechanism of H. pylori in the human gastric mucosa.

## MATERIALS AND METHODS

### Bacterial strains and growth conditions.

H. pylori strains used in this study are listed in Table 1. Strains TK1402, TK1029, and KR2003 are clinical isolates from Japanese patients (18, 51). TK1402CAMR1 and TK1402CAMR2 strains are CAM-resistant strains generated from TK1402 after treatment with CAM in our previous study (32). All strains were maintained at −80°C in brucella broth with 20% (vol/vol) glycerol and were cultured under microaerobic conditions at 37°C on brucella agar plates containing 7% horse serum (brucella-HS agar).

**TABLE 1:** Bacterial strains used in this study

Strain	Description	Source or reference
TK1402	Wild type	15
TK1029	Wild type	15
KR2003	Wild type	15
NCTC11638	Wild type	41
TK1402*alpB*::FLAG	*alpB*::FLAG Cm^r^	This study
TK1029*alpB*::FLAG	*alpB*::FLAG Cm^r^	This study
NCTC11638*alpB*::FLAG	*alpB*::FLAG Cm^r^	This study
TK1402Δ*alpB*	TK1402 derivative, Δ*alpB*::Km^r^	This study
NCTC11638Δ*alpB*	NCTC11638 derivative, Δ*alpB*::Km^r^	This study
TK1402Δ*alpB/alpB*_1402_	TK1402Δ*alpB*::TK1402 *alpB*, Cm^r^	This study
TK1402Δ*alpB/alpB*_11638_	TK1402Δ*alpB*::NCTC11638 *alpB*, Cm^r^	This study
TK1402Δ*alpB/alpB*_1029_	TK1402Δ*alpB*::TK1029 *alpB*, Cm^r^	This study
TK1402Δ*alpB/alpB*_2003_	TK1402Δ*alpB*::KR2003 *alpB*, Cm^r^	This study
TK1402Δ*alpB/alpB*_1402_V	TK1402Δ*alpB*::TK1402 *alpB* with TK1402 *alpB* variant region, Cm^r^	This study
TK1402Δ*alpB/alpB*_11638_V	TK1402Δ*alpB*::TK1402 *alpB* with NCTC11638 *alpB* variant region, Cm^r^	This study
TK1402Δ*alpB/alpB*_1029_V	TK1402Δ*alpB*::TK1402 *alpB* with TK1029 *alpB* variant region, Cm^r^	This study
TK1402Δ*alpB/alpB*_2003_V	TK1402Δ*alpB*::TK1402 *alpB* with KR2003 *alpB* variant region, Cm^r^	This study
NCTC11638Δ*alpB/alpB*_1402_	NCTC11638Δ*alpB*::TK1402 *alpB*, Cm^r^	This study

### Biofilm formation assay.

Biofilm formation was carried out as previously described (18). Briefly, sterilized glass coverslips (approximately 22 by 22 mm; 0.12- to 0.17-mm thickness; Matsunami Glass, Tokyo, Japan) were placed into 12-well microtiter plates. Each well was filled with 2 ml of brucella broth supplemented with 7% fetal calf serum (brucella-FCS broth) to allow adherence of H. pylori at the air-liquid interface. The biofilm formation was initiated by inoculating 10 μl of precultured cell suspension (approximately 5 × 10^5^ cells in brucella broth) into each well. The cultures were incubated under microaerobic conditions at 37°C for 3 days with shaking (80 to 100 rpm). After incubation, the coverslips were removed and washed with phosphate-buffered saline (PBS). The coverslips were then air dried and stained with crystal violet (CV) for 30 s. After being stained, the coverslips were rinsed with distilled water and then air dried. All dye associated with the biofilms was dissolved with 1 ml of ethanol, and 200-μl aliquots of the ethanol solutions were used to measure the absorbance at 594 nm with a microplate reader to determine the amount of biofilm formation.

### Construction of *alpB* mutant strains.

The *alpB* gene mutant strain was created by double-crossover homologous recombination via insertion of a kanamycin (Km) resistance determinant into the *alpB* gene of H. pylori strain TK1402 or NCTC11638. The upstream or downstream regions of each strain were amplified by PCR with specific pairs of primers (BF1/BR3tagKm for upstream and BF7tagKm/BR1 for downstream), with chromosomal DNA from H. pylori strains as a template (Table 2). The Km resistance gene cassette (*aph-A3*) was amplified by PCR with a specific primer pair (KmF/KmR) (Table 2), and the cassette was inserted into the upstream and downstream fragments of the target gene by using an overlap extension PCR method (52). The resulting up-Km-down fragment was transformed into wild-type TK1402 or NCTC11638 by natural transformation as previously described (53) to generate TK1402Δ*alpB* (TK1402 *alpB* mutant) and NCTC11638Δ*alpB* (NCTC11638 *alpB* mutant) strains (Table 1). The transformants were plated into brucella-HS agar supplemented with Km. The gene disruption was confirmed either by Southern blotting, PCR, or sequencing (data not shown).

**TABLE 2:** Primers used in this study

Primer	Nucleotide sequence^*a*^ (5′–3′)
BF1	CAACAGCTCACCTACTTGA
BR3tagKm	GGGTACCGAGCTCGAATTCTCATGTTTGTCTCTTCCCC
BF7tagKm	GGGGATCCTCTAGAGTCAAAAGCTCAAGGCCTTTTATAGG
BR1	AGTATCTTGGCCCTGCTTA
AF4	TTGCGCGCTCATCTTTAAC
BR5tagCm	CGACGACGGAATGGGATATTTAGAAGGCGTAGCCATAGA
BF6tagCm	CAGTTTGTCGCACTGATAAAAGCTCAAGGCCTTTTATAGG
BR6	GAATGGCTTTAAATTTTGTTTCAT
BF10	ATGAAACAAAATTTAAAGCCATTC
BR7	CGGATACAAATCAATCAAATTGC
BF11	GCAATTTGATTGATTTGTATCCG
BR8	GGTGTTGTTGTTGCCTTGAA
BF2	TTCAAGGCAACAACAACACC
BRFLAG	**TTACTACTTATCGTCGTCATCCTTATAATCAATATCATGATCTTTATAATCACCATCATGATCTTTATAATC**GAAGGCGTAGCCATAGAC
CmFFLAGtag	GGATGACGACGATAAGTAGTAAGTGGATAGATTTATGATATAATGAG
KmF	GAGCTCGGTACCCGGGTGA
KmR	GACTCTAGAGGATCCCCG
CmF	ATATCCCATTCCGTCGTCG
CmR	ATCAGTGCGACAAACTGGG
CmRKmintag	TGCTCCAGCCATCATGCCGATCAGTGCGACAAACTG
KmFin	CGGCATGATGGCTGGAGCA
BF2	TTCAAGGCAACAACAACACC
BR2	AATCCCACCCGCTTGGTT
Hp16SF	GAAGATAATGACGGTATCTAAC
Hp16SR	ATTTCACACCTGACTAT

a Tag sequence sites are underlined, and 3× FLAG sequences are shown in boldface.

### Construction of *alpB* restored strains.

**FIG 8:**
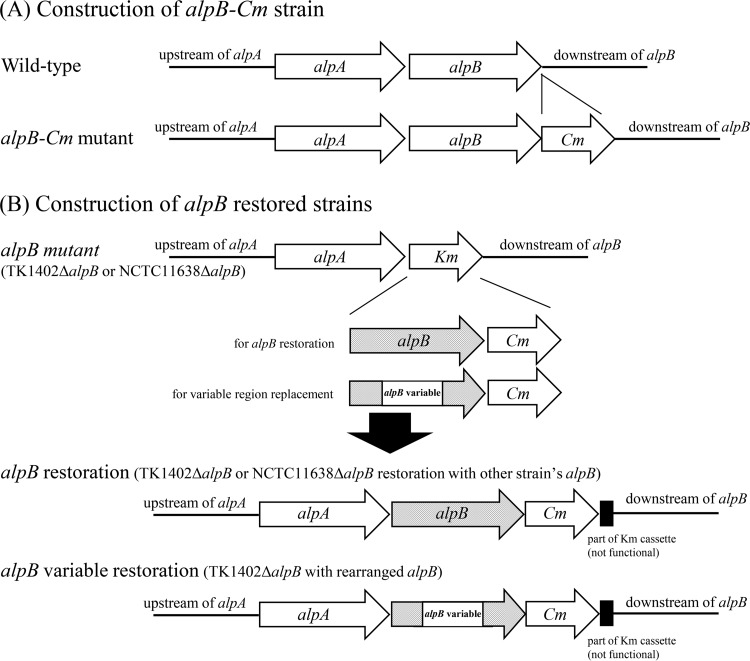
Schematic construction of the *alpB*-Cm mutant (A) and *alpB* restored (B) strains. All of the fragments were created with overlap extension PCR. These fragments were introduced into H. pylori strains by natural transformation.

For construction of *alpB* restored strains, we first constructed *alpB*-chloramphenicol acetyltransferase gene (*alpB*-Cm) cassette mutant strains with strains TK1402, NCTC11638, TK1029, and KR2003. Briefly, the *alpB* region containing the stop codon was amplified with forward primer AF4 and reverse primer BR5tagCm (Table 2). The downstream region of *alpB* was amplified with forward primer BF6tagCm and reverse primer BR1 (Table 2). The Cm cassette was amplified with primers CmF and CmR. The genomic DNA of *luxS* mutant strain HPKY08, described previously (54), was used as a template. The amplified cassette was inserted into the upstream and downstream fragments of the target gene by using an overlap extension PCR method (52), and the resultant fragment was transformed into H. pylori wild-type strains as described above (Fig. 8A). The transformants were confirmed by PCR or sequencing. The chromosomal DNA of the *alpB*-Cm mutant strain was extracted using a MagExtractor kit (Toyobo Co., Ltd., Osaka, Japan) according to the instructions of the supplier. The genomic DNA served as the template for constructing the *alpB* restored strain. Briefly, the upstream region of *alpB* was amplified with the forward primer BF1 and reverse primer BR6. Wild-type TK1402 or NCTC11638 was used as a template. The *alpB*-Cm region was amplified with primers BF10 and CmKmintag using genomic DNA of each strain's *alpB*-Cm mutant strain as a template. The downstream region was amplified with primers KmFin and BR1 using the genomic DNA of the TK1402Δ*alpB* or NCTC11638Δ*alpB* strain as a template. These amplified fragments were connected using the overlap extension PCR method and transformed into the TK1402Δ*alpB* or NCTC11638Δ*alpB* strain as described above to generate restored strains (the TK1402Δ*alpB/alpB*_1402_, TK1402Δ*alpB/alpB*_11638_, TK1402Δ*alpB/alpB*_1029_, TK1402Δ*alpB/alpB*_2003_, and NCTC11638Δ*alpB/alpB*_1402_ strains) (Table 1 and Fig. 8B). The strains were confirmed by antibiotic sensitivity (since the sequences of CmKmintag and KmFin primers are located inside the Km cassette, the restored strain exhibited resistance to Cm and sensitivity to Km) by PCR or sequencing. The *alpB* variable region-replaced restored strains (TK1402Δ*alpB/alpB*_1402_V, TK1402Δ*alpB/alpB*_11638_V, TK1402Δ*alpB/alpB*_1029_V, and TK1402Δ*alpB/alpB*_2003_V strains) were also constructed by a similar method using specific primer pairs (AF4/BR7 with TK1402 wild-type DNA as a template for upstream, BF11/BR8 for variable region insertion with each wild-type strain, and BF2/BR1 for downstream with TK1402 *alpB*-Cm DNA) (Table 1 and Fig. 8B). The replaced region is indicated by a box in Fig. S1 in the supplemental material.

### Construction of AlpB-FLAG strains.

To tag the chromosomal *alpB* gene with the DNA sequence encoding the triple FLAG sequence, overlap PCR was carried out. Briefly, the upstream region of the *alpB* gene was amplified using forward primer BF1 and reverse primer BRFLAG containing the 3′-terminal sequence without the stop codon of *alpB* but with a following sequence encoding triple FLAG epitopes, with the genomic DNA of each wild-type strain as a template. The downstream region containing the Cm cassette was amplified using CmFFLAGtag and BR1, with the genomic DNA of each *alpB*-Cm strain as a template. These fragments were connected using overlap extension PCR and transformed into wild-type strains TK1402, NCTC11638, and TK1029 as described above. The transformants were selected by Cm resistance and confirmed by PCR or by sequencing.

### Quantitative RT-PCR.

To analyze the expression of *alpB* mRNA, H. pylori strains were cultured in brucella-FCS broth for 1 or 2 days at 37°C. After washing of the cells with PBS three times, total RNA extraction was carried out using the RNeasy minikit (Qiagen GmbH, Hilden, Germany). The RNA samples were then treated with a Turbo DNA-free kit (Applied Biosystems, Foster City, CA) according to the directions of the supplier, and removal of DNA was confirmed by PCR with 16S rRNA-specific primers (Hp16SF/Hp16SR) (Table 2) (55). Reverse transcription was carried out with the PrimeScript RT reagent kit (TaKaRa Bio Inc., Shiga, Japan) according to the directions of the supplier. Real-time RT-PCR was performed with the cDNA samples with either 16S rRNA primers (Hp16SF/Hp16SR) (Table 2) or *alpB*-specific primers (BF2/BR2) (Table 2) with the SYBR premix *Ex Taq* (perfect real-time) kit (TaKaRa Bio Inc.) in an ABI PRISM 7500 real-time PCR system (Applied Biosystems). The final results were expressed as the levels of gene expression relative to that of the 16S rRNA gene.

### Preparation of proteins and Western blotting.

For preparation of protein samples, H. pylori strains were grown under microaerobic conditions at 37°C with shaking in brucella-FCS broth. After cultivation, the cells were collected by centrifugation (100,000 × *g* for 15 min). The collected cells were disrupted by a Minute bacterial total protein extraction kit (Invent Biotechnologies Inc., Plymouth, MN) according to the supplier's instructions. Preparation of OMV from the supernatant was performed as described previously (18). Briefly, H. pylori strains were grown in brucella-FCS broth for 24 h or 48 h. After cultivation, the cells were collected by centrifugation (10,000 × *g* for 15 min), and the resulting supernatants were filtered (low-protein-binding Durapore membrane, 0.45-mm polyvinylidene fluoride; Millipore, Bedford, MA) to remove the cells. The filtrates were centrifuged (40,000 × *g* for 2 h at 4°C), washed with PBS, and recentrifuged (40,000 × *g* for 2 h at 4°C). The pellets were resuspended in PBS supplemented with 0.2 M NaCl.

The samples were adjusted for protein concentration, treated with sodium dodecyl sulfate (SDS) loading buffer, including 5% 2-mercaptoethanol at 100°C for 5 min, and separated by polyacrylamide gel electrophoresis. The separated proteins were transferred to polyvinylidene difluoride membranes (Atto). After transfer, the membrane was blocked with 3% bovine serum albumin in PBS for 60 min at room temperature and incubated with monoclonal anti-FLAG M2 (Sigma) antibody to probe the FLAG tag at 37°C for 60 min. After washing with PBS containing 0.05% Tween 20 (PBS-T), peroxidase-labeled rabbit anti-mouse immunoglobulin (Dako A/S) was used as the secondary antibody. After washing with PBS-T, the blot was developed using ECL Plus (GE Healthcare Bio-Sciences).

### Mass spectrometry.

The 56-kDa protein band was excised from a Coomassie blue-stained SDS-PAGE gel and treated with trypsin. Digested protein was run through a mass spectrometer system (LTQ-Orbitrap Velos mass spectrometer) that was coupled with a direct nano-liquid chromatography system (DiNA; KYA Technologies). The Mascot search engine was used to access the NCBI bacterial database.

### DNA sequencing.

Genomic DNA of H. pylori strains was extracted using MagExtractor (Toyobo Co., Ltd., Osaka, Japan) according to the instructions of the supplier. The genomic DNA served as the template for PCR using *alpB* region-specific primer pairs (Table 2). Nucleotide sequences were analyzed directly for purified PCR products. Sequencing reactions were performed in a Bio-Rad DNA Engine Dyad PTC-220 Peltier thermal cycler using ABI BigDye Terminator V3.1 cycle sequencing kits with AmpliTaq DNA polymerase (FS enzyme) (Applied Biosystems, Foster City, CA) according to the instructions of the supplier. Single-pass sequencing was performed on each template with an ABI 3730xl sequencer (Applied Biosystems).

### AGS cell adherence.

An adhesion assay was carried out on TK1402, TK1402Δ*alpB*, TK1402Δ*alpB/alpB*_1402_, TK1402Δ*alpB/alpB*_1402_V, TK1402Δ*alpB/alpB*_11638_, and TK1402Δ*alpB/alpB*_11638_V strains using a CFU counting method. AGS human gastric cancer cells were cultured according to standard procedures; the cells were seeded into 12-well plates and grown to >90% confluence in F-12 medium supplemented with 10% FBS. H. pylori strains were cultured under microaerobic conditions at 37°C for 3 days with shaking in brucella-FCS broth. After cultivation, the optical density at 600 nm (OD_600_) of the cell culture was adjusted to 1.0, and adjusted cultures were mixed with equal amounts of F-12 medium. AGS cells were washed three times with PBS, and the H. pylori suspensions were added to the wells at a multiplicity of infection of 10. The mixtures were incubated in a CO_2_ incubator at 37°C for 2 h. After incubation, the wells were washed 3 times with PBS followed by addition of 1% saponin in PBS. After mixing with mechanical treatment, the mixtures were plated onto brucella-HS agar. After cultivation for 5 days, the CFU values of H. pylori were determined.

### Statistical analysis.

Statistical analysis was performed using the Mann-Whitney U test. *P* values of 0.05 or less were considered to indicate statistical significance.

## Supplementary Material

Supplemental material
