# Supplementary material for: Diversification of the AlpB Outer Membrane Protein of Helicobacter pylori Affects Biofilm Formation and Cellular Adhesion
Source: J Bacteriol. 2017 Feb 28;199(6):e00729-16. doi: 10.1128/JB.00729-16 (PMC5331671; doi:10.1128/JB.00729-16)
Supplement: Supplemental material [file supp_199_6_e00729-16__index.html]

Supplemental material 

# Diversification of the AlpB Outer Membrane Protein of Helicobacter pylori Affects Biofilm Formation and Cellular Adhesion

## Supplemental material

- Supplemental file 1 -

  Fig. S1 (Alignment of *H. pylori* AlpB amino acid sequences) and S2 (Two-dimensional structures of AlpB)

  PDF, 370K
