# Supplementary material for: Diversification of the AlpB Outer Membrane Protein of Helicobacter pylori Affects Biofilm Formation and Cellular Adhesion
Source: J Bacteriol. 2017 Feb 28;199(6):e00729-16. doi: 10.1128/JB.00729-16 (PMC5331671; doi:10.1128/JB.00729-16)
Supplement: Supplemental material [file JB.00729-16_zjb999094334s1.pdf]

```
1402    1:MKQNLKPFKMIKENLMTQSQKVRFLAPLSLALSLSFNQVGAEEDGGFMTFGYELGQVVQQ 60
1029    1:MKQNFKPFKMIKENLMTQSQKVRFLAPLSLALSLSFNQVGAEEDGGFMTFGYELGQVVQQ 60
2003    1:MKQNLKPFKMIKENLMTQSQKVRFLAPLSLALSLSFNQVGAEEDGGFMTFGYELGQVVQQ 60
11638   1:MKQNLKPFKMIKEDLMTQSQKVRFLAPLSLALSLSFNPVGAEEDGGFMTFGYELGQVVQQ 60
          ****.********.***********************.**********************

1402   61:VKNPGKIKAEELAGLLNSTTTNNTNINIAGTGGNVAGTLGNLFMNQLGNLIDLYPTLNTS 120
1029   61:VKNPGKIKAEELAGLLNSTTTNNTNINIAGTGGNVAGTLGNLFMNQLGNLIDLYPTLNTS 120
2003   61:VKNPGKIKAEELAGLLNSTTTNNTNINIAGTGGNVAGTLGNLFMNQLGNLIDLYPTLNTS 120
11638  61:VKNPGKIKAEELAGLLNSNTTNNTNTNIAGTGGNVAGTLGNLFMNQLGNLIDLYPTLNTS 120
          ******************.******.**********************************

1402  121:SIHQCGSGSTNGATTAATSSSTSNSPCFQGNLSLYNEMVSSIKTLSQDLKNNIFQGNNNT 180
1029  121:SIHQCNSGS---SSTAAAT--TSSSPCFQGNLSLYNEMVSSIKTLSQDLKNNIFQGDNNT 175
2003  121:NIHQCNSGS-NGSTTAAAT--TSSSSCFQGNLSLYNEMVSSIKTLSQDLKNNIFQGNNNT 177
11638 121:SISQCSATN-SGSSGATAAAATSNNPCFQGNLALYNKMVGSIKTLSQNISKNIFQGNNNT 179
          .*.** ...  .. .*..   **  ..******.***.**.*******....*****.***

1402  181:TSANLSNQLSELNTASVYLTYMNSFLNANNQAGGIFQNNTNQKYGNGVTAQQIAYVLKQA 240
1029  176:TSANLSNQLSELNTASVYLTYMNSFLNANNQAGGIFQNNTNQAYGNNVTAQQIAYVLKQA 235
2003  178:TSANLSNQLSELNTASVYLTYMNSFLNANNQAGGIFQNNTNQAYGNGVTAQQIAYVLRQA 237
11638 180:TSQNLSNQLSELNTASVYLTYMNSFLNANNQAGGIFQNHTNQAYGNGVTAQQIAYILKQA 239
          **.***********************************.***.***.********.*.**

1402  241:SITMGPSGNGGAAGAFLDAALAQHVFNSANAGNDLSAKEFTSLVQNIVNTSNNALTLANN 300
1029  236:SITMGPSGNSGAAGAFLDAALAQHVFNSANAGNDLSAKEFTSLVQNIVNTSNNALTLANN 295
2003  238:SITMGPSGNGGAAGAFLDAALAQHVFNSANAGNDLSAKEFTSLVQNIVNTSQNALTLANN 297
11638 240:SITMGPSGDSGAAAAFLDAALAQHVFNSANAGNDLSAKEFTSLVQNIVNNSQNALTLANN 299
          ********. ***.***********************************.* ********

1402  301:ANISNSTGYQVSYGGNIDQARSTQLLNNTTNTLAKVTALNNELKANPWLGNFAAGNSSQV 360
1029  296:ANISNSTGYQVSYGGXIDQARSTQLLNNTTNTLAKVAALNNELKANPWLGNFAAGNSSQV 355
2003  298:ANISNSTGYQVSYGGNIDQARSTQLLNNTTNTLAKVTALNNELKANPWLGNFAAGNSSQV 357
11638 300:ANISNSTGYQVSYGGHIDQARSTQLLNNTTNTLAKVTALNNELKANPWLGNFAAGNSSQV 359
          *************** ********************.***********************

1402  361:NAFNGFITKIGYKQFFGENKNVGLRYYGFFSYNGAGVGNGPTYNQVNLLTYGVGTDVLYN 420
1029  356:NAFNGFITKIGYKQFFGENKNVGLRYYGFFSYNGAGVGNGPTYNQVNLLTYGVGTDVLYN 415
2003  358:NAFNGFITKIGYKQFFGENKNVGLRYYGFFSYNGAGVGNGPTYNQVNLLTYGVGTDVLYN 417
11638 360:NAFNGFITKIGYKQFFGENKNVGLRYYGFFSYNGAGVGNGPTYNQVNLLTYGVGTDVLYN 419
          ************************************************************

1402  421:VFSRSFGSRSLNAGFFGGIQLAGDTYISTLRNSPQLASRPTATKFQFLFDVGLRMNFGIL 480
1029  416:VFSRSFGSRSLNAGFFGGIQLAGDTYISTLRNSPQLASRPTATKFQFLFDVGLRMNFGIL 475
2003  418:VFSRSFGSRSLNAGFFGGIQLAGDTYISTLRNSPQLASRPTATKFQFLFDVGLRMNFGIL 477
11638 420:VFSRSFGSRSLNAGFFGGIQLAGDTYISTLRNSPQLASRPTATKFQFLFDVGLRMNFGIL 479
          ************************************************************

1402  481:KKDLKSHNQHSIEIGVQIPTIYNTYYKAGGAEVKYFRPYSVYWVYGYAF            529
1029  476:KKDLKSHNQHSIEIGVQVPTIYNTYYKTGGAEVKYYRPYSVYWVYGYAF            524
2003  478:KKDLKSHNQHSIEIGVQIPTIYNTYYKAGGAEVKYYRPYSVYWVYGYAF            526
11638 480:KKDLKSHNQHSIEIGVQIPTIYNTYYKAGGAEVKYFRPYSVYWVYGYAF            528
          *****************.*********.******* *************
```

Alignment of AlpB amino acid sequences in *H. pylori* strains used in this study (1402:TK1402, 1029:TK1029, 2003:KR2003, 11638:NCTC11638). The variable region corresponding to amino acid positions 121 to 146 of AlpB is shown in underline. The replaced region for the construction of TK1402Δ*alpB/alpB*$_{1402}$V, TK1402Δ*alpB/alpB*$_{11638}$V, TK1402Δ*alpB/alpB*$_{1029}$V and TK1402Δ*alpB/alpB*$_{2003}$V is shown in a box.

Fig. S1

(A) TK1029

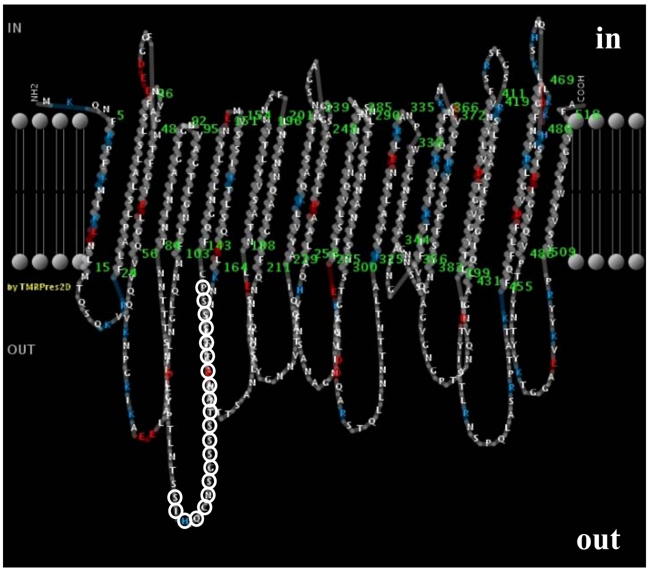


(B) KR2003

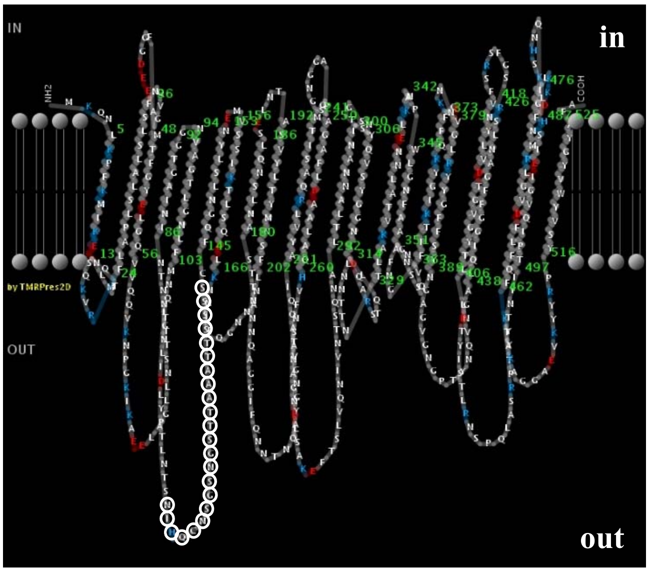


(C) TK1402Δ*alpB/alpB*$_{11638}$

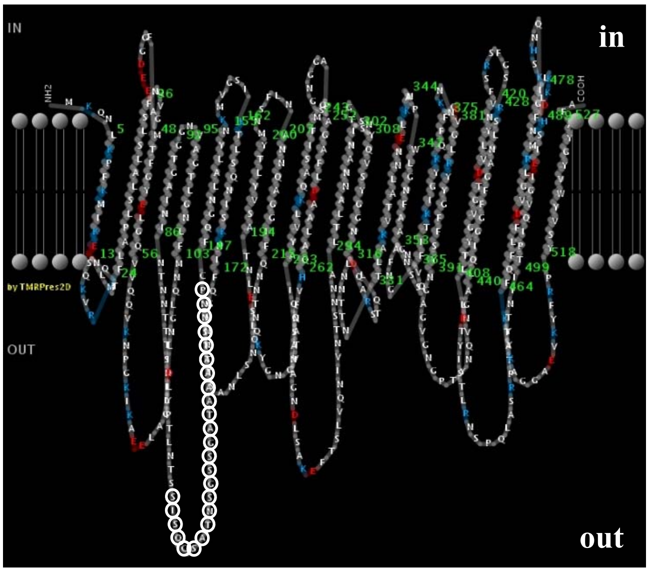


(D) TK1402Δ*alpB/alpB*$_{1029}$

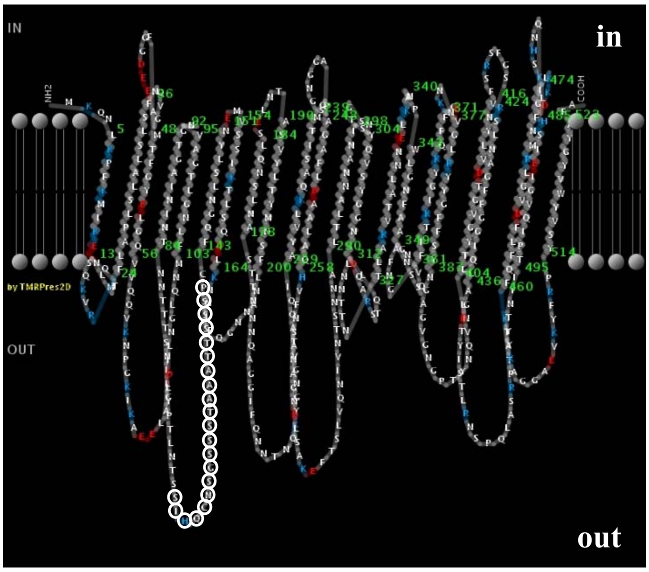


(E) TK1402Δ*alpB/alpB*$_{2003}$

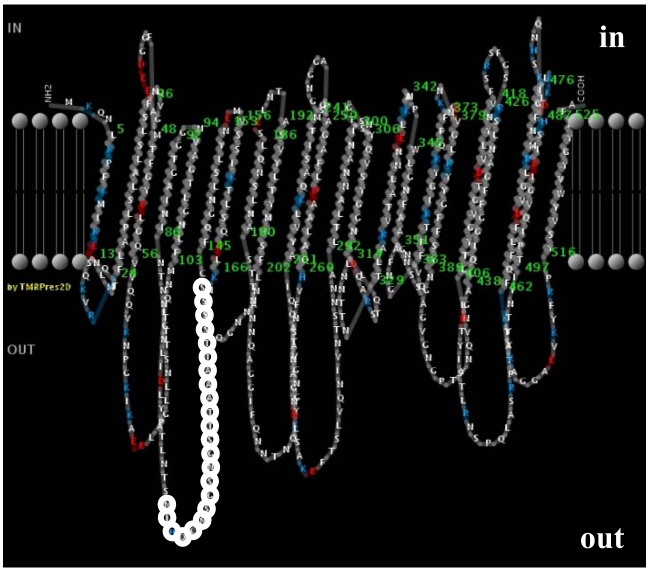


Two-dimensional structure of AlpB from strains TK1029 (A), KR2003 (B), TK1402Δ*alpB/alpB*$_{11638}$ (C), TK1402Δ*alpB/alpB*$_{1029}$ (D), and TK1402Δ*alpB/alpB*$_{2003}$ (E), based on the prediction by PRED-TMBB. The amino acids of the variable region are shown by white circles. Strains TK1402Δ*alpB/alpB*$_{11638}$, TK1402Δ*alpB/alpB*$_{1029}$ and TK1402Δ*alpB/alpB*$_{2003}$ were restored strains derived from the TK1402 *alpB* mutant restored with NCTC11638, TK1029 or KR2003 *alpB*, respectively.

Fig. S2
